# Protein expression plasticity contributes to heat and drought tolerance of date palm

**DOI:** 10.1007/s00442-021-04907-w

**Published:** 2021-04-21

**Authors:** Andrea Ghirardo, Tetyana Nosenko, Jürgen Kreuzwieser, J. Barbro Winkler, Jörg Kruse, Andreas Albert, Juliane Merl-Pham, Thomas Lux, Peter Ache, Ina Zimmer, Saleh Alfarraj, Klaus F. X. Mayer, Rainer Hedrich, Heinz Rennenberg, Jörg-Peter Schnitzler

**Affiliations:** 1 Research Unit Environmental Simulation, Institute of Biochemical Plant Pathology, Helmholtz Zentrum München, 85764 Neuherberg, Germany; 2 Institute of Forest Sciences, Chair of Ecosystem Physiology, Georges-Köhler-Allee 53/54, 79110 Freiburg, Germany; 3 Institute of Forest Sciences, Chair of Tree Physiology, Georges-Köhler-Allee 53/54, 79110 Freiburg, Germany; 4 Research Unit Protein Science, Helmholtz Zentrum München, 85764 Neuherberg, Germany; 5 Plant Genome and Systems Biology, Helmholtz Zentrum München, 85764 Neuherberg, Germany; 6 Institute for Molecular Plant Physiology and Biophysics, University Würzburg, 97070 Würzburg, Germany; 7 College of Sciences, King Saud University, P.O. Box 2455, Riyadh, 11451 Saudi Arabia; 8 School of Life Sciences, Technical University Munich, Munich, Germany; 9 Center of Molecular Ecophysiology, College of Resources and Environment, Southwest University No. 2, Tiansheng Road, Beibei District, 400715 Chongqing, People’s Republic of China

**Keywords:** Abiotic stress, Isoprene, Proteomics, Photosynthesis, Phoenix dactylifera

## Abstract

Climate change is increasing the frequency and intensity of warming and drought periods around the globe, currently representing a threat to many plant species. Understanding the resistance and resilience of plants to climate change is, therefore, urgently needed. As date palm (*Phoenix *dactylifera) evolved adaptation mechanisms to a xeric environment and can tolerate large diurnal and seasonal temperature fluctuations, we studied the protein expression changes in leaves, volatile organic compound emissions, and photosynthesis in response to variable growth temperatures and soil water deprivation. Plants were grown under controlled environmental conditions of simulated Saudi Arabian summer and winter climates challenged with drought stress. We show that date palm is able to counteract the harsh conditions of the Arabian Peninsula by adjusting the abundances of proteins related to the photosynthetic machinery, abiotic stress and secondary metabolism. Under summer climate and water deprivation, these adjustments included efficient protein expression response mediated by heat shock proteins and the antioxidant system to counteract reactive oxygen species formation. Proteins related to secondary metabolism were downregulated, except for the *P. dactylifera* isoprene synthase (PdIspS), which was strongly upregulated in response to summer climate and drought. This study reports, for the first time, the identification and functional characterization of the gene encoding for PdIspS, allowing future analysis of isoprene functions in date palm under extreme environments. Overall, the current study shows that reprogramming of the leaf protein profiles confers the date palm heat- and drought tolerance. We conclude that the protein plasticity of date palm is an important mechanism of molecular adaptation to environmental fluctuations.

**Supplementary Information:**

The online version contains supplementary material available at 10.1007/s00442-021-04907-w.

## Introduction

Climate change is expected to increase the frequency and intensity of high temperatures and dry spells (IPCC 2013; Arnell et al. 2019; Baldwin et al. 2019; Kornhuber et al. 2019). Heatwaves and prolonged drought episodes are threats for many plant species, including several agricultural and forest plants. However, during evolution, some plants have survived remarkable seasonal variations of temperature and soil water availability by developing complex adaptation strategies to maintain metabolic homeostasis (Bréda et al. 2006) although the underlying mechanisms are still poorly understood. To ensure human food security and to remodel threatened forests, a deeper understanding of plants' adaptability and resilience to the consequences of global warming is urgently required for the development of smart agricultural systems and the implementation of successful forest management.

Date palm (*Phoenix dactylifera* L.) is naturally distributed across arid/semi-arid environments typical of Middle East (Shabani et al. 2012). It is one of the oldest crop species and, because of its economic importance, cultivations have been extended to Australia, Asia, Africa, and the Americas (Tengberg 2012). The variety of climate conditions in which the date palm can grow shows that it tolerates adverse climate conditions: adapted to broad temperature range (12.7–27.5 °C as averages), date palm withstands frost and hot periods of − 5 and + 50 °C (Chao and Krueger 2007) and long drought episodes (Du et al. 2019). Therefore, *P*. *dactylifera* provides a good model for dissecting molecular and physiological key processes that able plants to cope with extreme climate conditions (Arab et al. 2016).

Proteins respond to abiotic stresses at transcriptional, post-transcriptional, translational, and post-translational levels. Hence, adjustments of protein expression and modification can assist *P*. *dactylifera* in managing extreme environmental changes. An in-depth understanding of the tolerance mechanisms of date palm to its native climate habitat may help unravel the strategies that plants evolved to successfully withstand a wide range of environmental conditions similar to those expected in other parts of the globe under the effects of climate change.

Under severe drought stress, most plants react by closing their stomata to limit transpiration water loss. In turn, leaf internal carbon dioxide (CO_2_) concentrations drop, impairing net CO_2_ assimilation (*A*) (Brunner et al. 2015). This restriction of CO_2_ fixation with a simultaneous continuing light reaction of photosynthesis often leads to the formation of reactive oxygen species (ROS) causing oxidative stress due to electron leakage to oxygen molecules (Rennenberg et al. 2006; Lee et al. 2012). In general, the detoxification of ROS is achieved by the use of efficient antioxidants such as ascorbate and glutathione and their regeneration in the Foyer-Halliwell-Asada cycle (Foyer and Noctor 2011). Enzymes involved in the antioxidative response include the superoxide dismutase (SOD), catalase (CAT), glutathione reductase (GR), L-ascorbate peroxidase (APX), monodehydroascorbate reductase (MDHAR), and dehydroascorbate reductase (DHAR) (Noctor et al. 2012; Bartwal et al. 2013; Nievola et al. 2017). Some metabolites such as carotenoids, polyphenols, and proline also possess antioxidant properties, although they cannot be recycled easily. Biosynthesis of the volatile organic compound (VOC) isoprene, however, is known to counteract oxidative stress and protects the photosynthetic apparatus. Isoprene helps leaves against abiotic stresses, especially during episodes of extremely high temperatures and drought (Sharkey et al. 2008; Loreto and Schnitzler 2010). Although the mechanism is not yet fully understood, it is shown that isoprene production affects the antioxidant system leading to a reduction in the level of reactive oxygen species (ROS) (Velikova et al. 2004, 2012, 2014, 2015; Vickers et al. 2009). It also affects the secondary metabolic pathways of phenols, fatty acids, tocopherols and carotenoids, some of them are also involved in the quenching of harmful radicals (Behnke et al. 2010; Way et al. 2013; Kaling et al. 2014; Ghirardo et al. 2014). Compared to non-volatile molecules, the volatility of isoprene allows rapid penetration into membranes, diffusion through plant organelles and no need to be recycled, which may help plants to withstand acute heat stress (Behnke et al. 2007, 2013). Isoprene emission is strongly light-dependent, and its formation occurs in the chloroplasts of some, but not all, plant species, including numerous woody plants (Monson et al. 2013). Palm species such as oil and date palm are strong isoprene emitters (Benjamin et al. 1996; Wilkinson et al. 2006) and the study of isoprene emission is climate-relevant, as it participates in the formation of ozone, organic nitrates, aerosol formation and consumption of hydroxyl radicals in the atmosphere (Fuentes et al. 2000; Poisson et al. 2000; Ghirardo et al. 2016; Kiendler-Scharr et al. 2009).

In the present study, we investigated the molecular and biochemical mechanisms of tolerance in the young date palm plants to high temperatures and mild-to-severe water shortage using simulated environmental conditions. These experiments were performed in climate chambers of the eco-/phytotron at Helmholtz Zentrum München, which provides a realistic simulation of climate and solar radiation (Seckmeyer and Payer 1993; Döhring et al. 1996; Thiel et al. 1996; Kozovits et al. 2005; Ghirardo et al. 2020; Roy et al. 2021). To this end, we acclimatized the plants to the summer and winter climate prevailing in Saudi Arabia and studied photosynthesis, VOC emissions and the leaf proteome upon summer drought (SD) and winter drought (WD) conditions, and compared with well-irrigated summer control (SC) and winter control (WC) conditions. These harsh climates, characteristic of the natural habitats of date palm, will likely occur in future in other regions under the effects of global warming.

In previous studies, partially employing the same experimental approach, we focused on leaf photosynthesis and stomatal conductance (Kruse et al. 2019), changes of leaf metabolites (Du et al. 2019), antioxidative system, and fatty acid metabolism (Arab et al. 2016). Here, we study the plant volatile emission and the adjustments of the leaf proteome composition in response to heat and drought. In addition, based on bioinformatics analyses of genomic, transcriptomic and proteomics data, we report here for the first time the identification of the *P*. *dactylifera* isoprene synthase gene (*PdIspS*) and present the functional characterization of the respective enzyme key in abiotic stress tolerance.

## Materials and methods

### Plant material and experimental setup

Two-year-old date palm (*Phoenix dactylifera* L.) plants were purchased from a commercial supplier ('Der Palmenmann', Bottrop, Germany) and transferred into 3.3-L pots filled with a peat-soil-sand mixture (3:1:7 v/v/v) and 10 g of Osmocote fertilizer (16-9-12%, N-P-K). Plants were grown two months under greenhouse conditions (photoperiod of 12 h; 25/15 °C, 20/30% rh, day/night) and irrigated once per week (*c*. 150–200 ml per pot) before they were transferred to the four climate chambers of the eco-/phytotron at Helmholtz Zentrum (Kruse et al. 2019; Ghirardo et al. 2020; Roy et al. 2021).

Two of the four climate chambers were used to simulate the Saudi Arabian summer conditions, the other two to simulate the winter climate (see below for details). Each chamber was equipped with four sub-chambers, each hosting 15 plants. Per chamber, plants in two sub-chambers were exposed to water deprivation, whereas plants in the other two sub-chambers were kept well-watered as controls (*n* = 4 sub-chambers per treatment).

During the first week, plants were acclimated in the climate chambers under gradually changing experimental parameters. We simulated the winter and the summer climate in Alahsa, Saudi Arabia, using a 10-years of average of temperatures in 2003–2012, observed in winter (21.12.-21.03) or summer (21.06–21.09; Supplementary Information, Fig. S1, for details, Kruse et al. 2019). Data on relative humidity were only available for 2013.

Winter and summer day climates were maintained throughout the duration of the experiment (7 weeks). Compared to winter, the photoperiod was four hours longer in summer, while the maximum irradiance at midday was similar, leading to a daily light integral of 20.6 (summer) and 15.6 (winter) mol m^−2^ day^−1^ PPFD (Supplementary, Fig. S1). Besides the day length, environmental conditions strongly differed in temperature: average noon temperature peaked at 40 °C in summer and at 25 °C in winter, with a day/night temperature amplitude of 20 °C. The relative humidity dropped in winter conditions from 80% during the night to 18% at noon and from 35 to 8% in summer.

**Fig. 1:**
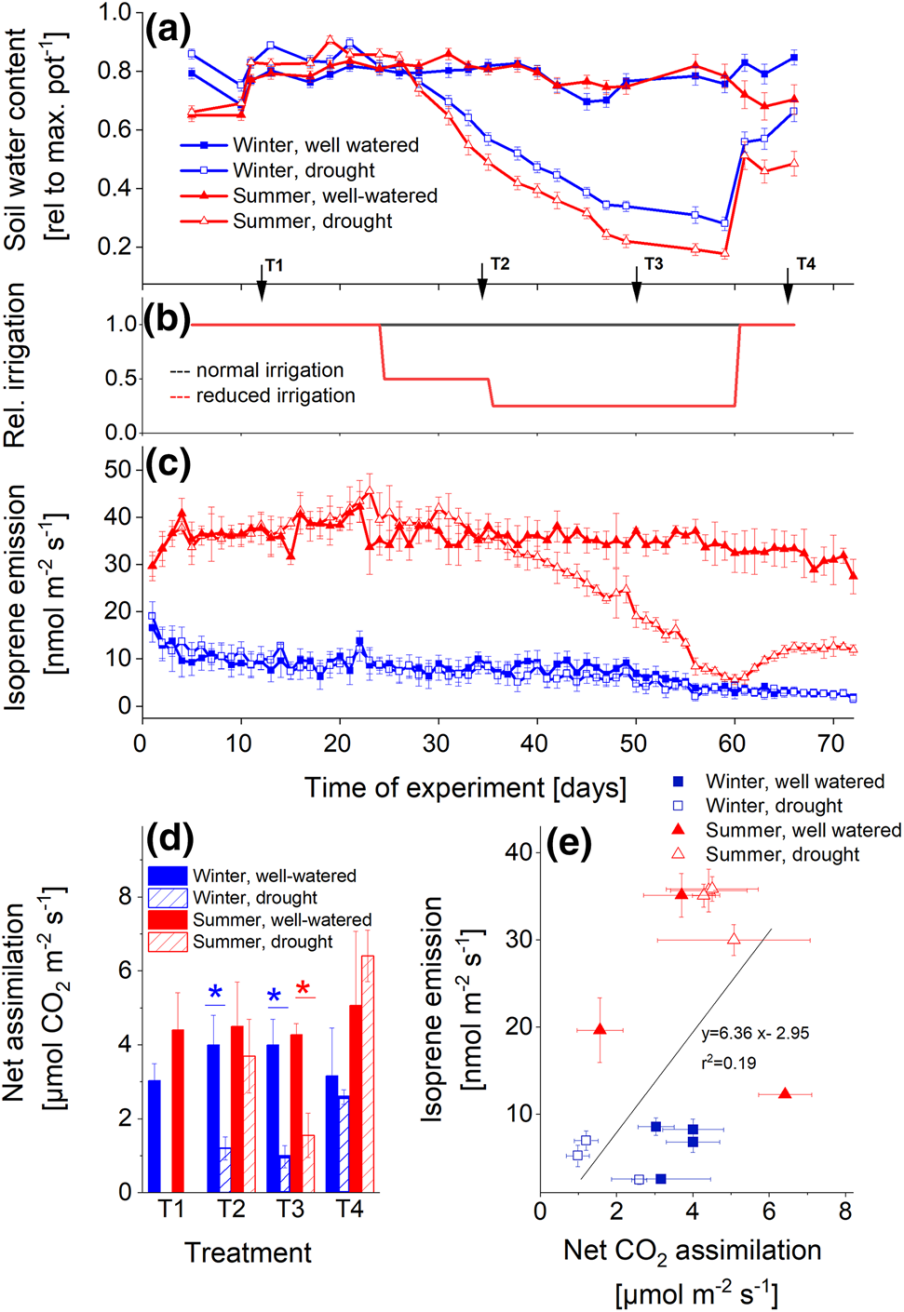
Effects of climate and drought on isoprene emission and net CO_2_ assimilation. **a** Relative soil water content of plant substrate; **b** relative irrigation level during the experiment; **c** isoprene emissions at midday (1 h mean at 12 noon); **d** net CO_2_ assimilation; **e** relationship between net assimilation and isoprene emission. T1–T4 indicates sampling times under pre-stress (T1), mild (T2), and severe (T3) water deprivations, and following re-watering (T4). Black arrows indicate the timepoints of leaf sampling for the proteomic analysis. Data shown are means ± SE (*n* = 4 replicates); *p < 0.05

To progressively lower the soil water content (SWC), the irrigation was reduced 50% relative to SC and WC on the days of experiment 24–27 and to 25% after further seven days. The effects of SD and WD on date palms were studied either continuously or at four different time points (T1-4, see Fig. 1), which included pre-treatment (T1), mild (T2), and severe stress (T3), as well as after re-watering (T4). The SWC was measured with a soil moisture sensor (ML3 Thetaprobe, Delta-T, UK) and given as percentage of the maximum reading observed for the pot during the complete experiment.

### Gas-exchange and VOC analyses

Gas-exchange of CO_2_ was measured under standard conditions ([CO_2_]: 400 ppm; light: 1000 µmol m^−2^ s^−1^ PPFD; temperature: 30 °C) on fully expanded leaves by infrared gas analyzers (GFS-3000, Walz, Effeltrich, Germany) as described elsewhere (Kruse et al. 2019). Three plants per sub-chamber were randomly chosen and treated as technical replicates for the measurements of CO_2_ gas-exchange. In total and per treatment, we measured 12 plants from four different sub-chambers (*n* = 4). Gas-exchange measurements were conducted at midday (11:00 am–1:30 pm).

The emissions of VOC in the climate chambers were monitored by online mass spectrometry throughout the whole experiment. The high-sensitivity proton-transfer-reaction quadrupole mass-spectrometer (PTR-QMS) was operated as previously described (Ghirardo et al. 2010, 2011; Kreuzwieser et al. 2014) and in combination with the chamber system of the EUS eco-/phytotron (Vanzo et al. 2015). Detailed information of the VOC system and the purification of the inlet air is given elsewhere (Ghirardo et al. 2020).

Sampling and analysis of VOCs were achieved by gas chromatography-mass spectrometry (GC–MS) as before (Duan et al. 2020). Collection of VOCs was performed simultaneously to CO_2_ gas-exchange measurements by diverting an aliquot of the air (4.5 L collected using an airflow of 100 ml min^−1^) from the cuvette outlet into GC–MS sampling tubes (Gerstel, Mülheim, Germany) filled with Tenax/Carbotrap/Carboxen 569 (20/30/40 mg; Supelco, Bellafonte, PA).

### Harvest of plant material

Leaves from five different plants were harvested at time 1:30 pm at the end of T1, T3, and T4, and fresh weight was determined. For the determination of dry mass, leaves were dried for three days at 65 °C.

### Label-free analysis of date palm leaf proteome using Progenesis LC–MS

Proteomic analysis was performed as before (Monson et al. 2020). All LC–MS/MS spectra were used for peptide identification with Mascot (v2.5.1). The annotation and functional classification were achieved based on the *P. dactylifera* genome annotation (38,570 predicted protein models; Hazzouri et al. 2019) and the Swissprot Green Plant protein database (38,396; https://www.uniprot.org/) as described in Miloradovic van Doorn et al. (2020). Five biological replicates were analyzed per treatment. The mass spectrometry data have been deposited to the ProteomeXchange Consortium via the PRIDE (Perez-Riverol et al. 2019) partner repository (identifier: PXD021666).

### Proteomic mapping to MapMan functional categories (BINs) and pathway analysis

For each sample comparisons (SC/WC, SD/SC, WD/WC), protein identifiers and calculated log2 fold ratios were imported into MapMan (v3.6.0RC1, https://mapman.gabipd.org/) (Thimm et al. 2004; Usadel et al. 2005, 2009). Maps were created based on the Arabidopsis database, and the corresponding protein orthologs were searched on SMARTBLAST (https://blast.ncbi.nlm.nih.gov/smartblast). The program compares protein sequences in databases and returns the accessions of all the proteins from different plant species found with the respective statistical significance of matches. Among these, the respective Arabidopsis orthologs with the highest identity were used in MapMan.

### Identification and primary sequence analyses of the putative IspS gene from *Phoenix dactylifera*

To identify *P. dactylifera* orthologs and close paralogs of genes encoding proteins known to possess isoprene synthase (IspS) activity, BLAST sequence similarity searches were conducted against *P. dactylifera* predicted protein models and genome sequence assembly GCA_000413155.1 (NCBI Bioprojects PRJNA396270) using *Populus tremula* CAC35696, *P. alba* ADG96473.1, *P. fremontii* AEK70967.1, *Eucalyptus globulus* BAF02831.1, *Melaleuca alternifolia* AAP40638.1 and *Arundo donax* ASF20076.1, *Casuarina equisetifolia* BAS30549.1 and *Humulus lupulus* ACI32638.1 *IspS* genes as queries. The resulting sequences were aligned to the reference *IspS* sequences using MUSCLE (Edgar 2004) and analyzed for the presence of conserved motifs and diagnostic tetrad residues described elsewhere (Sharkey et al. 2013; Li et al. 2017) using Mesquite (Massidon and Maddison 2018). Amino acid alignments of the candidate genes were searched to detect exact matches to the *P. dactylifera* peptides-markers of putative terpene synthase (TPS). To reconstruct the N- and C-termini of the partial *IspS* sequence XP_008779509.1, we identified close orthologs of this gene from other monocots from the NCBInr database and used these sequences to screen genome data for the *P. dactylifera* cultivars Khalas and Khanizi (NCBI Bioprojects PRJNA396270 and PRJNA322046), respectively. Contigs resulting from this screening were assembled in a single pseudo-scaffold, further verified and corrected using RNA-seq data available for *P. dactylifera* from the NCBI Short Read Archive (SRA) as described in Supplementary MM1. The CDS was translated using Mesquite and the cleavage site of the *IspS* plastid-targeting peptide was predicted with TargetP v1.1 (Emanuelsson et al. 2000).

### *Phoenix dactylifera *IspS CDS cloning, expression in *E. coli* and in vitro enzymatic activity assay

The CDS sequence encoding mature *PdIspS* (1695 bp after removing the chloroplast transit peptide) was edited and optimized for expression in *E. coli* using the GeneArt portal software (Invitrogen). The attB1- and attB2-Express motifs were added to the N- and C-termini of the CDS sequence, respectively. The resulting sequence was synthesized, cloned into the Gateway donor vector pENTR221 and subsequently subcloned into the Gateway destination vector pDEST17 (Invitrogen) by Life Technologies GmbH. Cloning efficiency was verified using restriction enzymes *XbaI* and *HindIII* and the in-gel insert size verification protocol.

The *PdIspS* was expressed in the chemically competent *Escherichia coli* cells BL21(DE3) (ThermoFisher Scientific, Darmstadt, Germany). Chemical transformation of the competent cells and protein expression were conducted according to the manufacturer protocol.

Protein extracts of heterologously expressed PdIspS were obtained as previously described (Schnitzler et al. 2005), and PdIspS enzyme activity was assayed in vitro*,* according to Mayrhofer et al. (2005). Synthesized isoprene was measured by headspace analysis using PTR-QMS (Ghirardo et al. 2010, 2014). Date palm IspS activities were determined as described in Supplementary MM2 using 5 mM of the substrate dimethylallyl diphosphate (DMADP) for isoprene biosynthesis or by 5 mM geranyl diphosphate (GDP) for monoterpenes.

The temperature response curve of PdIspS activity was modeled by the Arrhenius equation:

1 $$k= {A\mathrm{e}}^{-\frac{Ea}{RT}},$$

where *k* is the enzymatic rate constant, *A* is the frequency factor of the process, *E*_a_ is the activation energy (J mol^–1^), R is the gas constant (8.314463 J mol^–1^ K^–1^), and *T* is the absolute temperature (K).

### Statistical analysis

The four subchambers per treatment served as the units of replication (i.e., *n* = 4) for isoprene and photosynthesis analyses.

To test for differences in VOC profiles, data of relative VOC emission rate (peak area m^−2^ s^−1^) of drought-stressed, non-stressed, and re-watered plants grown in summer and winter climate were subjected to principal component analysis (PCA) using MetaboAnalyst 3.0 (Xia et al. 2015; Chong et al. 2019; Pang et al. 2020). Data were subjected to logarithmic transformation, centered and scaled to unit variance to conform to a normal distribution and ensure equal weighing of all compounds.

Statistical differences of proteomics data were analyzed using PCA and Orthogonal Partial Least Square regression (OPLS) analyses using SIMCA-P (v13.0.0.0, Umetrics, Umeå, Sweden) as described elsewhere (Vanzo et al. 2015). PCA was calculated on normalized protein intensities (X-variables) after log_10_ and unit-variance transformations. The results were validated by full cross-validation (CV) (Eriksson et al. 2008) using a 95% confidence level. Additionally, discriminant proteins were independently tested for significant difference between the comparisons SC/WC, SD/SC, WD/WC using one-way ANOVA (*p* < 0.05, FDR of 5%) (SigmaPlot v11.0, Systat Software, Erkrath, Germany).

Statistically significant BINs were tested using the Wilcoxon rank-sum test implemented in MapMan after Benjamini Hochberg correction (FDR) of 5%. The hypergeometric distribution test (*p* < 0.05) was performed for over/underrepresentation analysis of BINs in the different protein classes (Goffard and Weiller 2006).

## Results

### Drought differently affects photosynthesis and isoprene emissions in date palm

The VOC emissions and the CO_2_ gas-exchange were studied from date palms growing under simulated winter and summer climate of Alahsa, Saudi Arabia, challenged by drought stress and compared to well-watered control conditions (Fig. 1). Plants under SC showed significantly (*p* < 0.001, ANOVA) higher isoprene emission rates (~ 40.1 ± 2.0 nmol m^−2^ s^−1^ at noon, under *T* = 39.4 ± 0.4 °C and light of 640 µmol m^−2^ s^−1^; Fig. 1c) than plants growing under WC (8.5 ± 1.6 nmol m^−2^ s^−1^), whereas differences in photosynthetic net CO_2_ assimilation were small (Fig. 1d). Water deprivation caused a substantial decline in SWC in both climates but impaired the net CO_2_ assimilation rates (*A*) in WD but not in SD under mild drought (T2, Fig. 1d), when the decrease of SWC was 54–67% (Fig. 1a). Isoprene emissions remained unaffected in plants experiencing mild drought under both summer and winter climates compared to their respective well-watered controls (Fig. 1b). Under severe drought (T3) and compared to controls, *A* was strongly reduced in both SD and WD, but isoprene emissions decreased significantly only in SD (*p* < 0.05, ANOVA). Isoprene emission remained unaffected in WD, even when SWC was less than 30%. Upon re-watering of the plants, the SWD recovered, isoprene emission rates increased again in SC, *A* fully recovered (T4) in both climates and dry/fresh weight ratios increased (Supplementary Fig. S2). The high emission of isoprene in SC and its slighter decrease in SD, despite a quick decline in photosynthesis, suggests an involvement of isoprene biosynthesis to assist leaves under heat/drought stress.

**Fig. 2:**
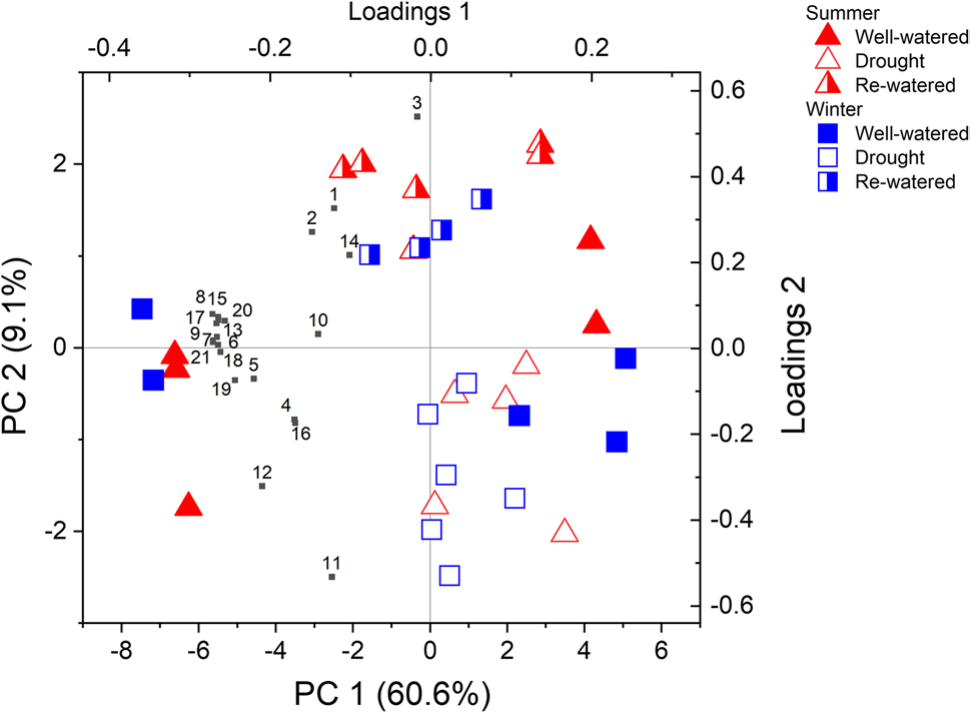
PCA biplot of date palm VOC emissions. Data depict samples collected from plants under drought (open symbols) and control (solid) conditions, or under drought recovery (half-open/solid), exposed to summer (red) or winter (blue) climate. The explained variance (in %) and the number of principal components (PC) are reported in x- and y-axes. Numbers reflect the compound IDs given in Supplementary Table S1

Emissions of stress-induced VOCs are molecular markers of physiological stress. We investigated date palm’s response to heat/drought stress by additionally collecting air samples for GC–MS analysis and analyzed by PCA. Besides isoprene, date palm emitted twenty additional plant VOCs (Supplementary Table S1), and these emissions changed under drought, as seen by the separation of drought from controls/re-watered samples in the significant principal component (PC) 1 and 2 (Fig. 2). Most of the VOC emissions positively correlated to well-watered/re-watered conditions, meaning that emissions decreased under severe drought (T3). Indicative of molecular oxidation, the oxygenated volatiles acetaldehyde (#1 in Fig. 2) and ethanol (#2 in Fig. 2) were specifically induced upon re-watering (Supplementary Table S1).

### Climate fluctuation affects the leaf proteome broader than soil water deprivation

**Fig. 3:**
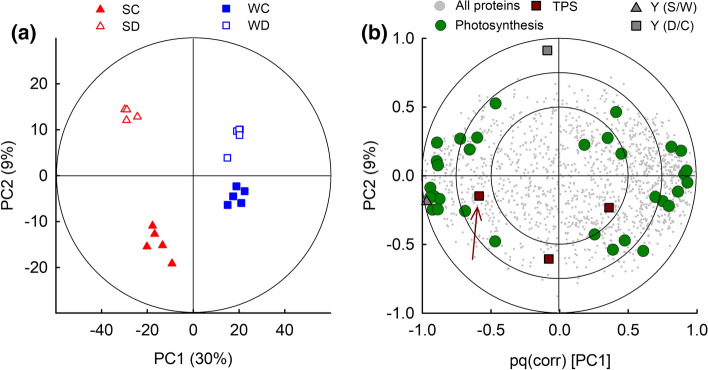
Global effects of climate and drought on date palm proteome. **a** Two-dimensional score plot of OPLS proteome analysis. The ellipse indicates OPLS tolerance (Hotelling’s *T*^2^) with *α* = 0.05. **b** OPLS loading plot (correlation-scaled to 1). The outer/inner ellipses indicate 100/75% of explained variance. The circles are the X-loadings (protein abundances) and the triangle and squares are the Y-loadings for the climates (S/W) and for the treatment (D/C) variables, respectively. *S* summer, *W* winter, *C* control, *D* drought, *PC* principal component. Arrow points to the putative terpene synthase (TPS) referred in the text (accession number Q5UB07/XP_008779509.1, Supplementary Table S2)

Climate and soil water deprivation caused clear physiological responses in date palms, such as the reduction of net CO_2_ assimilation and isoprene emission (Fig. 1). We investigated in detail the quantitative and qualitative leaf proteomics changes in extracts of date palm leaves collected under severe drought stress (T3) of the comparison SC/WC, SD/SC, and WD/WC. To this end, we quantitatively measured 1520 proteins identified using SwissProt Green Plant database and characterized the variation in protein expressions by a multivariate statistical approach based on OPLS (Fig. 3; Model fitness: *r*^2^ (*x*) = 38%, *r*^2^ (y) = 100%, *r*^2^ = 90.7%, and *q*^2^ (cumulative) = 65% using two predictive components. RMSEE (root mean square error of estimation) = 0.0909 (S/W), 0.225 (D/C); RMSEcv (root mean square error of CV) = 0.132 (S/W), 0.394 (D/C). *p* = 2.9∙10^–6^ (S/W), *p* < 0.05 (D/C)). The proteome of plants cultivated in SC was significantly (*p* < 0.001, CV-ANOVA) different from the protein composition in leaves of plants grown in WC. The comparison between SD/SC and WD/WC further showed that drought also led to significant changes (*p* < 0.05) in the leaf proteome. However, the influence of climate was greater than that of soil water deprivation, as shown by the 30% of the explained total variance in the PC1, which explains the separation of samples with respect to climate. In comparison, PC2, which describes the drought treatment, explains only 9% of the total variance. Because the OPLS aims to find the plane in the multivariate space along the difference between the sample groups are maximized, the greater distance between SD/SC than WD/WC indicate that date palm adjusted the proteome to a larger degree under hot conditions of summer than winter climate to withstand drought stress. This represents a quantitative analysis of the proteome-wide protein expression plasticity of date palm under extreme climate and soil water contents.

**Fig. 4:**
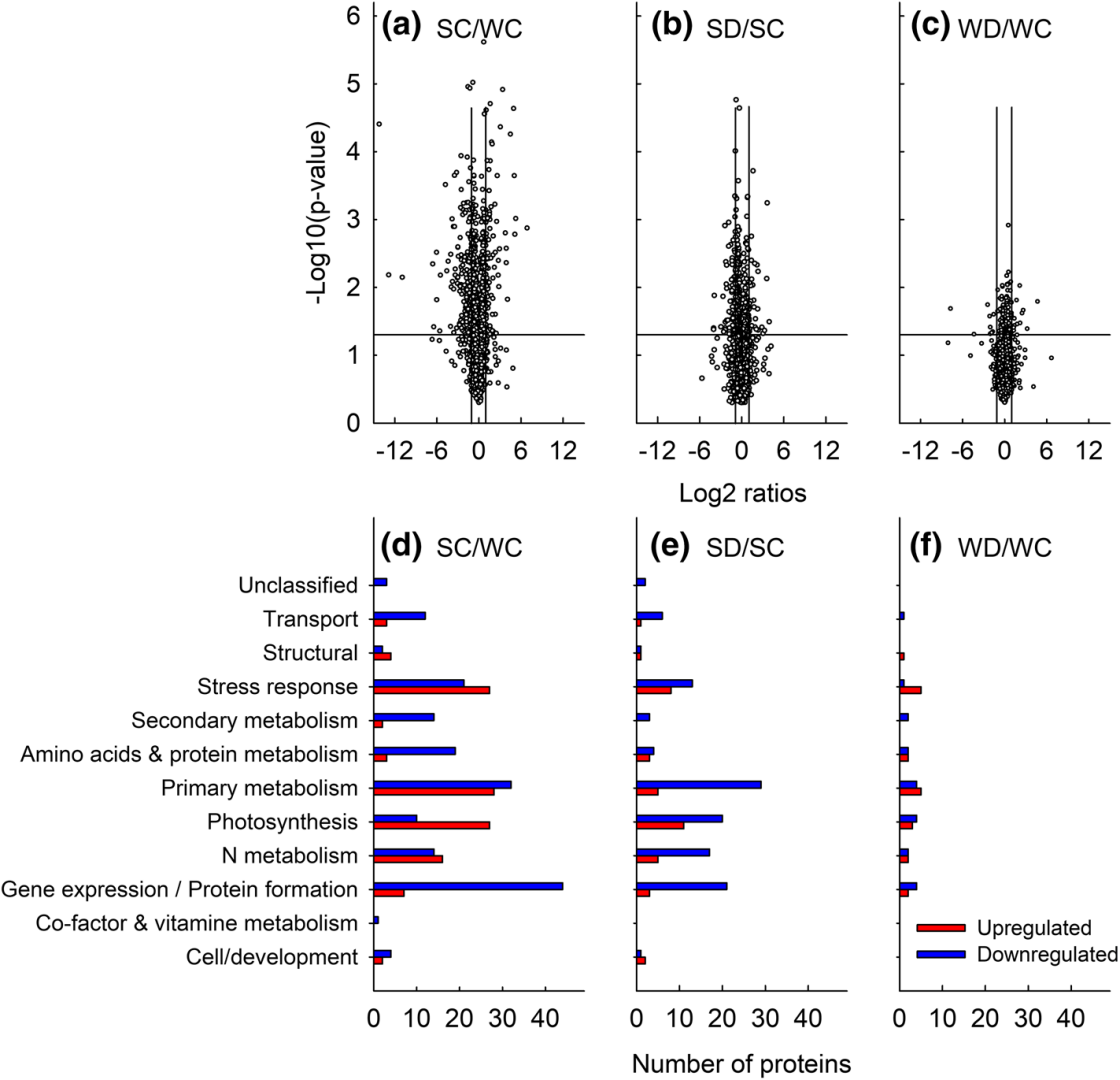
Proteomic changes following climate adaptation and drought. **a–c** Volcano plot showing the relative changes of protein abundance in date palm leaves (FC, log2 of fold change) compared with the measure of statistical significance (− log10 [*p* value, ANOVA]). Vertical lines indicate a FC of ± 1 and the horizontal line indicates the significance level of *p* < 0.05. **d**–**f** The number of proteins significantly (*p* < 0.05) present in low (blue bars) or high (red bars) abundances in date palm leaves as affected by climate (summer or winter) and water availability (drought or well-watered controls). Low or high abundance of proteins was counted when FC were <  − 1 or > 1, respectively. The proteins were grouped based on their putative biological function. *SC* summer-control, *SD* summer-drought, *WC* winter-control, *WD* winter-drought

**Fig. 5:**
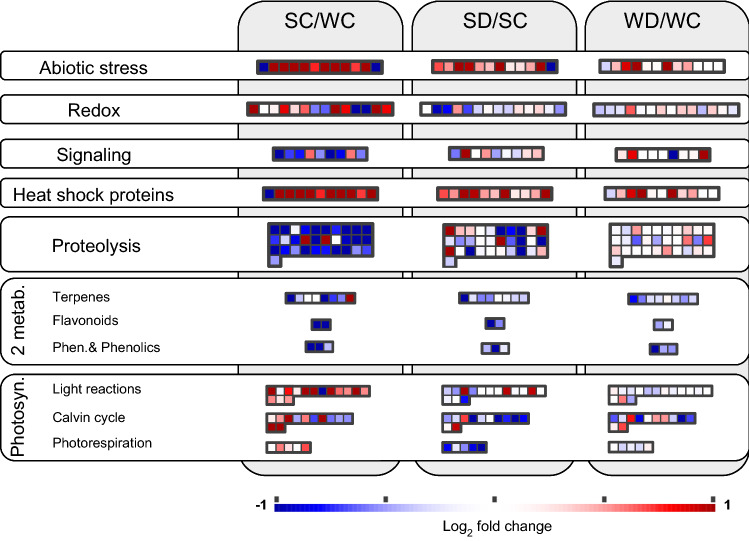
MapMan visualization of major proteomic changes. The map was created using MapMan (Usadel et al. 2005) and the corresponding Arabidopsis protein homologs using the significant protein expression changes given in Supplementary Table S2. Protein expression changes (squares) in the corresponding biosynthetic pathways, significantly different in either the comparison SC/WC, SD/SC, WD/WC are colored according to their log2 of fold ratios. *SC* summer-control, *SD* summer-drought, *WC* winter-control, *WD* winter-drought, *2 metab* secondary metabolism, *Photosyn* photosynthesis, *Phen* phenylpropanoids. *p* < 0.05 for each individual protein

Specifically, the expression of 295 proteins was significantly different in the foliar proteome of date palm acclimated to summer and winter climate, 64 related to photosynthesis (Fig. 3b, Supplementary Table S2). Far fewer proteins, i.e., 156 (31 related to photosynthesis) and 40 (7 related to photosynthesis) were differentially regulated in the comparisons SD/SC and WD/WC, respectively (Supplementary Table S2). Among those significant changes, we depicted the most strongly upregulated (log2 of fold changes (FC) of > 1) or downregulated (FC < -1) proteins (Fig. 4) and visualized the overall significant proteomic changes using MapMan, a tool to map proteins in functional categories (Fig. 5, Supplementary Table S2). Compared to WC, the heat of SC led to a remarkable (FC > 1) upregulation of proteins involved in primary metabolism (28), stress response (27) and photosynthesis (27), and a downregulation (FC < 1) of those involved in gene expression and protein formation (44), amino acid and protein metabolic processes (22), and secondary metabolism (14) (Fig. 4). Drought caused a more general downregulation of proteins. In respect to protein function, the processes of photosynthesis, abiotic stress, redox homeostasis, proteolysis, and secondary metabolites were significantly changed (*p* < 0.05, hypergeometric test; Fig. 5). Within these general adjustments, we studied in more detail (below) the individual proteins that were most affected under heat (comparison SC/WC) and soil water limitation (SD/SC and WD/WC).

### Summer heat acclimation is achieved by remodeling the photosynthesis-related proteome and by inducing stress-related proteins

We first investigated in detail the protein adjustments caused by climate in plants acclimated to summer and compared to plants grown in winter climate (comparison SC/WC). The protein abundances of the photosynthetic light and dark reactions were significantly upregulated. In particular, the content of eight proteins related to ribulose-1,5-bisphosphate carboxylase/oxygenase (Rubisco) and 2 ATP synthases increased (Supplementary Table S2). Another three proteins of chlorophyll metabolism, two related to the thylakoid membrane, and some central proteins of the photosystem II reaction center and the electron transport chain (ETC) such as, e.g., ubiquinol oxidase (GenBank accession number XP_008785033.1) were also more abundant in chloroplasts in SC than in the cooler WC (Supplementary Table S2). In contrast to these highly regulated proteins and indicative of lower demand of nutrient and energy transport inside the cell, proton pump H^+^-ATPase integrated into the plasma membrane (PM) was strongly downregulated (Q42556, FC = − 4.39), concomitant to a lower abundance of the mitochondrial adenosine diphosphate (ADP)/adenosine triphosphate (ATP) carrier (XP_008795699.1, FC = − 0.71).

The hot temperatures of SC induced the expression of several proteins involved in acclimation processes to abiotic stress. Notably, we found 16 heat shock proteins (Hsps), molecular chaperones crucial in thermotolerance, and four involved in regulating the redox homeostasis. The plastidic metalloprotein [Cu–Zn] SOD (XP_008813737.1), capable of quenching superoxide radical by production of H_2_O_2_ to mitigate oxidative stress, was strongly upregulated in SC/WC (FC = 1.88, adj. *p* value = 2.89E−03). Consistently, both APX (XP_008783664.1), which reduces H_2_O_2_ to H_2_O using L-ascorbate, and thiol-disulfide oxidoreductase (TDO, XP_008785058.1) that may participate in various redox reactions, were found significantly upregulated (FC, adj. *p* value: APX = 0.84, 0.011; TDO = 0.64, < 0.05). In contrast, GR (XP_008789436.1) was downregulated (FC = − 1.04, adj *p* value < 0.01). We also observed that the chloroplastic methionine sulfoxide reductase (MSR, XP_008784388.1), which restores protein activities by catalyzing the reduction of methionine sulfoxide to methionine, was upregulated (FC > 0.5, adj *p* value < 0.05) in plants grown under hot conditions.

Proteins involved in the secondary metabolism of terpenes, phenylpropanoids, phenols, and flavonoids were downregulated. Also, proteins involved in proteolysis and signaling were mainly downregulated (#Q338C0, FC = − 6.43). Only a putative terpene synthase (TPS; XP_008779509.1) was found upregulated (FC = 1.57).

### Drought amplifies proteomic differences of hot summer temperatures

We further investigate soil water deprivation effects by comparing the samples SD/SC and WD/WC (Fig. 4 and 5). Under summer climate, drought stress intensified proteomic differences caused by hot temperatures (seen from the comparison SC/WC), except for proteins involved in redox reactions and photosynthesis, which mainly decreased significantly. Upon water deprivation in SD/SC, 117 proteins were downregulated and 39 upregulated.

Under the cooler conditions of winter climate, drought-induced protein expression changes were less pronounced than in summer but affected a similar subgroup of proteins. The WD/WC comparison resulted in 20 downregulated and 20 upregulated proteins. Interestingly, the same upregulated and downregulated proteins in WD/WC were found in SD/SC, suggesting that the same subgroups of proteins were involved in drought acclimation regardless of temperature (Supplementary Table S2).

The methylation of protein and DNA has profound effects on enzyme and gene regulation. The abundance of the putative adenosylhomocysteinase (AdoHcyase, XP_008776857.1), crucial for the modulation of methyltransferase activity in cells, was strongly positively correlated with drought (VIP = 1.8, FC = 1.63; adj *p* value = 0.0196). In agreement with the negative effects of drought on assimilation and indicative of a decreased availability of energy and lower demand for active movement of ions and nutrients, an NADP-dependent glyceraldehyde-3-phosphate dehydrogenase (XP_008794414.1), two ATP carriers (P27081, XP_008795699.1), and a magnesium protoporphyrin involved in photosynthesis (XP_008778346.1,) were downregulated (FC < − 0.5, adj. *p* value < 0.05).

### *Phoenix dactylifera* IspS gene identification from proteomics data

Isoprene is important in protecting the photosynthetic apparatus from abiotic stresses, yet isoprene synthase (IspS) and its encoding gene (*IspS*) are not described in date palm so far. Therefore, we searched the IspS with the help of proteomic data. First of all, we correlated climate and soil water deprivation with proteins involved in photosynthetic processes and terpene production and found peptides of three potential terpene synthases (TPS, Fig. 3b). One of these proteins (Q5UB07) shows high homology to the tricyclic synthase TPS4 in *Medicago truncatula* and its protein content correlated positively with the high isoprene emissions in summer climate (Fig. 1, 3). These features made the protein an excellent candidate for the discovery of IspS.

**Fig. 6:**
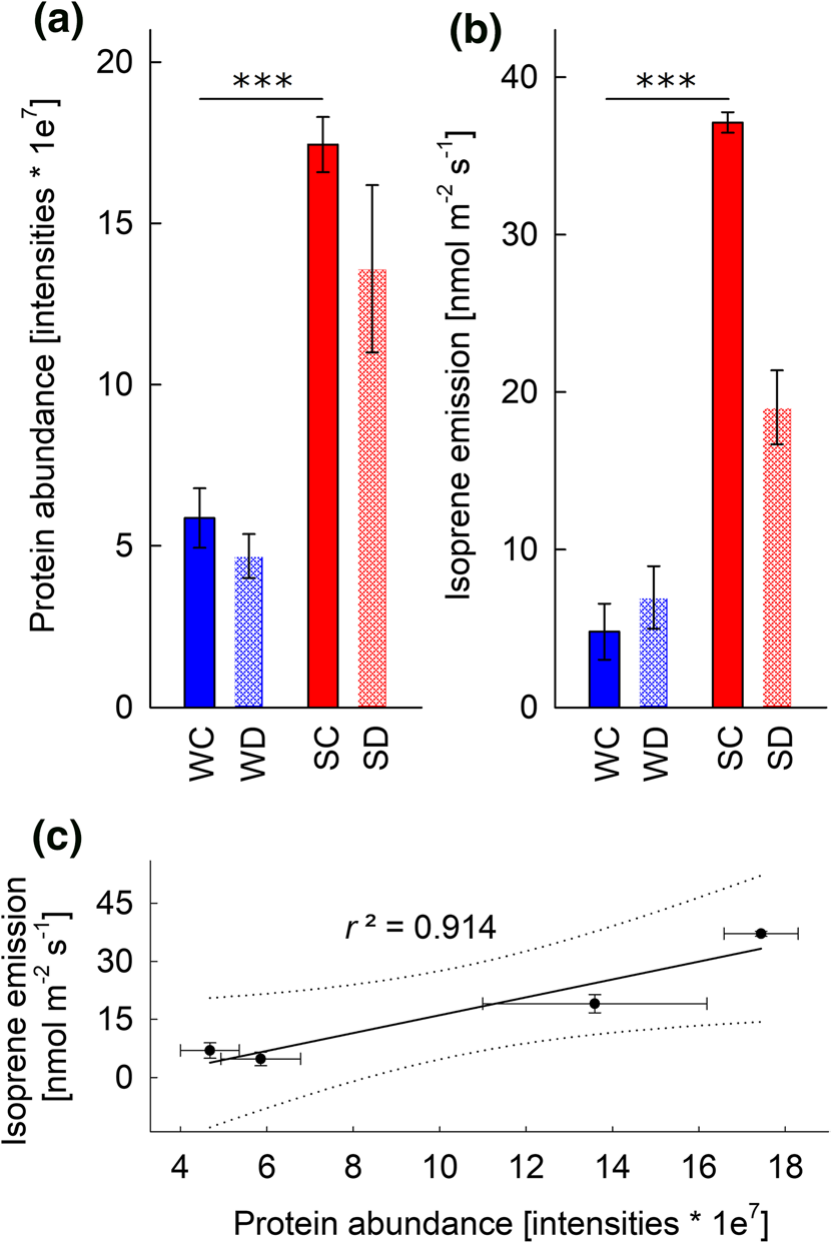
Correlation between protein levels and isoprene emissions. **a** Protein abundance (MS intensities of XP_008779509.1) and **b** isoprene emissions under simulated winter (in blue) and summer (in red) climate in Saudi Arabia and upon severe drought stress (white patterns). **c** Linear correlation between protein level and isoprene emissions. Dot lines depict confidence intervals of 95%. **a**–**c** Data were collected under T3 (severe drought stress). *SC* summer-control (red), *SD* summer-drought (red/white), *WC* winter-control (blue), *WD* winter-drought (blue/white). Data shown are means ± se of 4 (isoprene emissions) and 5 (protein levels) independent replicates

Screening *P. dactylifera* predicted protein models for sequences homologous to known isoprene synthases identified three candidate genes: XP_008775412.1, XP_017699994.1, and XP_008779509.1. Only one of these proteins, XP_008779509.1, a partial TPS, contains the first three residues of the diagnostic IspS tetrad F(V/S)F(N/S) (Supplementary Fig. S3; Sharkey et al. 2013; Li et al. 2017), and matched to the peptide-marker for one out of ten candidate proteins (LCNDLATSSAELER; Table S2). The presence of the diagnostic *IspS* tetrad (Supplementary Fig. S3) and the correlation of the protein expression with the climate differences of isoprene emission (Fig. 6) suggest that XP_008779509.1 was a strong candidate for the *IspS* gene in the *P. dactylifera* genome. XP_008779509.1 is a partial TPS, from which both N- and C-terminal ends of the *IspS* gene are missing. We reconstructed, therefore, the complete CDS of the putative *PdIspS* (Supplementary Figs S4-5) using genomic and RNA-seq data of the two date palm cultivars Khalas and Khanizi as described in Supplementary MM1. The putative *PdIspS* consists of 7 exons encoding 585 amino acid residues (1755 bp). The first 21 amino acids represent a putative chloroplast transit peptide (TargetP probability 72%). The *PdIspS* has a 16 amino acid long extension at its C-terminus relative to other plant *IspS* genes. Among functionally characterized IspS, the putative *PdIspS* showed the highest sequence similarity to *A. donax IspS* (52% amino acid sequence identity). The mature *PdIspS* of date palm consists of the conserved functional TPS motifs DDXXD, DTE/NSE, and RXR (Supplementary Figs S3-4).

### *Phoenix dactylifera* IspS functional characterization

**Fig. 7:**
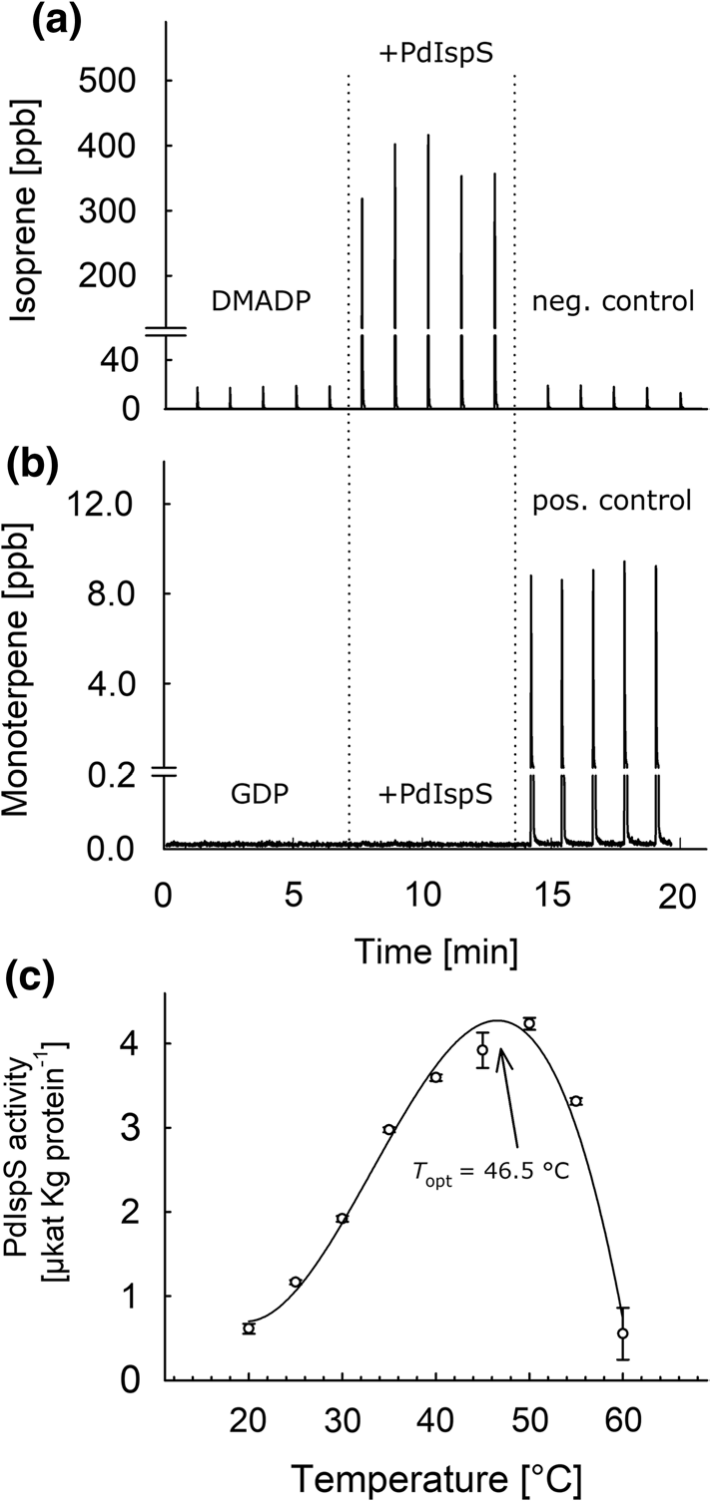
Functional characterization of PdIspS. Production of **a** isoprene with DMADP and **b** monoterpene with GDP following expression of PdIspS in *E. coli*, protein extraction and headspace analysis. Negative controls in **a** were performed using the protein extracts from *E. coli* transformed with an empty vector construct; positive controls in **b** were performed with pure monoterpene standard. **c** Temperature dependence of PdIspS enzyme activity (means ± se of five replicates). Data were fitted by the cubic polynomial function; optimum temperature (*T*_opt_) is the temperature when the fitted enzyme activity is at its maximum

The function of the putative *PdIspS* gene was demonstrated by heterologous expression of the corresponding mature protein in *E. coli*, followed by protein extraction and incubation of protein extracts with either the IspS substrate DMADP, or the monoterpene synthases substrate GDP as control (Fig. 7a-b). Mass spectrometric analysis revealed that the enzyme indeed produced isoprene, as seen by its formation in the presence of DMADP (Fig. 7a). As expected, we observed a small chemical degradation of DMADP to isoprene (Brüggemann and Schnitzler 2002) at amounts comparable to products formed by *E. coli* protein extracts transformed with the empty expression vector (negative control). Complementary analysis demonstrated that PdIspS has no monoterpene synthase activity (specifically, tricyclene synthase, Supplementary Table S2), as seen from the inability of the enzyme to convert GDP into a monoterpene.

Temperature response of isoprene emission mirrors this of IspS activity under saturating substrate concentrations (Rasulov et al. 2010). The analysis of PdIspS between 20 and 60 °C showed a typical enzyme activity profile of IspS with a maximum enzyme velocity (ranging between 0.3–2.3 µkat kg protein^−1^) at approximately 50 °C (Fig. 7c), suitable for plants growing under the hot summer conditions of the Arabian Peninsula. The relationship between temperature and enzyme activity can be well-described by the Arrhenius equation at temperatures from 20–50 °C indicating that the enzyme protein becomes denatured at higher temperatures. The activation energy (*E*_*a*_) of PdIspS was 50.3 kJ⋅mol^−1^.

## Discussion

Plants have evolved complex mechanisms to withstand harsh climates. Native to the Middle East, *P. dactylifera* provides an excellent model for studying complex mechanisms of plant adaptation. It grows under high temperatures and light intensities and is remarkably drought-tolerant. Combining climate chamber experiments with proteomics and VOC emission analyses, we showed a comprehensive picture in the adaptation of the date palm leaf proteome to naturally occurring climatic conditions of Saudi Arabia. The protein expression plasticity of the date palm contributed to the plant acclimation to a large fluctuation of environmental conditions. We related changes in the protein expression patterns observed under the seasonal climatic extremes to physiological processes such as photosynthesis and the emission of VOCs. As proteins are the functional macromolecules in cells, adjustments at protein expression level helped plants maintaining homeostasis of fundamental metabolic processes such as seen in photosynthesis and were instrumental in achieving cellular stress resistance under environmental changes. Our data suggest that the one underlying mechanism of date palm’s tolerance to heat and drought is the remarkable plasticity of its proteome.

### Proteomic adjustments counterbalance the adverse effects of heat and drought on photosynthesis

Heat and drought are among the most harmful and common abiotic stressors. They inhibit metabolic processes and damage key components of photosynthesis, such as ETC of photosystem II (damaging PSII), energy production (ATPase), and CO_2_-fixation (Rubisco) (Lu and Zhang 2000; Zhang et al. 2009). Typically, the consequences of heat and drought are stomatal closure and reduction of photosynthesis rates, which are then restored upon return to lower temperature or water availability. In the present study, analysis of CO_2_-assimilation (*A*) showed that the physiological responses of date palm were similar to those of woody temperate plant species under moderate stress: drought stress-induced partial stomatal closure but upon re-watering photosynthesis fully recovered (see also Kruse et al. 2019). Analysis of VOCs indicated that date palm incurred, to some extent, cellular stress as emissions of typical stress-induced oxygenated compounds under recovery were indicative of molecular oxidation (Niinemets et al. 2014). However, date palm did not experience a critical heat/drought stress condition, as photosynthesis fully recovered and we did not observe any phenotypic signs of injury such as leaf chlorosis/necrosis of withering leaves or damage of PSII following excessive formation of ROS that would have led to membrane leakiness and eventually cellular death. Sensing external abiotic stress stimuli was depicted by increasing abundances of proteins involved in signaling, in functioning as regulatory factors, protein transporters, G-proteins and calcium ion binding (Supplementary Table S2). Soil water deprivation under summer climate increased AdoHcyase, an important enzyme of the S-adenosyl-L-methionine (SAM) cycle that generally increases in leaves in response to drought stress (Wang et al. 2016). AdoHcyase is involved, among others, in the epigenetic process of thermomemory (Zhang 2018; Lamelas et al. 2020), which may help date palm "remembering" the thermal stress to next generations.

The full recovery of physiological parameters and similar *A* in plant acclimated to summer and winter climates indicated that date palm was fully able to withstand the heat/drought applied. We investigated, therefore, the mechanisms behind this extraordinary ability to cope with such harsh environmental conditions. As previously shown, increasing enzyme activities of the antioxidant system are important for maintaining the redox homeostasis (Arab et al. 2016). Here, the profound adjustments at protein levels of the photosynthetic machinery and abiotic stress-related proteins, secondary metabolism, and protein metabolisms, as discussed below, indicate a complex protein reprogramming in date palm leaves to confer heat/drought tolerance.

### Adjustment of leaf proteome and resilience of isoprene emission to support photosynthesis

The proteome of date palms acclimated to summer climate showed, compared to winter climate, a much higher expression of proteins related to the light and dark reactions of photosynthesis concomitant with strong isoprene emissions. These proteome-wide adjustments supported photosynthesis and are consistent with the metabolic reprogramming of chloroplasts under heat stress (Sharkey 2005; Wang et al. 2018). Summer climate induced the expression of proteins related to Rubisco, chlorophyll metabolisms, PSII and ETC. Among the most affected proteins, we found a higher abundance of the chloroplastic protein magnesium protoporphyrin IX monomethyl ester cyclase, which is involved in chlorophyll biosynthesis during the metabolism of porphyrin-containing compounds and catalyze the formation of the isocyclic ring (Tottey et al. 2003).

Heat/drought stress also caused the increase of ATP synthases and downregulation of the H^+^-ATPase integrated in PM. ATP synthases are crucial in energy transduction and alleviation of stress; they confer tolerance to drought in peanut and Arabidopsis (Zhang et al. 2008; Kottapalli et al. 2009) and their increases are consistent to those observed in date palm under severe drought (El Rabey et al. 2016). The H^+^-ATPases are proton-symport for the transport of sugars and amino acids across the PM (Morsomme and Boutry 2000). The downregulation of H^+^-ATPases in our study was indicative of lower demand for active movements of nutrients. In agreement to lower H^+^-ATPase abundances, the mitochondrial ADP/ATP transporter was downregulated. Such adenosine transports are necessary for regular cell metabolism since the ADP/ATP cycle provides energy for the metabolite reactions (Klingenberg 2008).

The hot summer climate also increased the expression of PdIspS and, respectively, the leaf emissions of isoprene. High emissions were maintained under mild drought stress despite the decline in photosynthesis. In our study, isoprene emissions were affected by water deprivation only after 14 days and later than photosynthesis (see also Kruse et al. 2019). The effects of drought on isoprene emissions agree with the severity of the stress: emission decreases under severe but not mild drought, when it becomes partially sustained by non-photosynthetic carbon supply (Pegoraro et al. 2004, 2006; Brilli et al. 2007; Perreca et al. 2020). The resilience of emissions under limited photosynthetic capacity suggests a role of isoprene under drought. Finally, we demonstrated a significant correlation between PdIspS expression and isoprene emission under a broad spectrum of environmental changes. This is valuable information that may help process-based modeling approach (Grote et al. 2006, 2009) to improve estimates of regional and global isoprene emissions under drought conditions.

Isoprene can protect the photosynthetic apparatus from abiotic stress (see review Monson et al. 2021) as it is effective against cellular oxidative stress occurring during drought, strong light, or ozone exposure (Loreto and Velikova 2001; Affek and Yakir 2002; Velikova et al. 2004; Vickers et al. 2009; Behnke et al. 2009). Using transgenic non-isoprene emitting poplars, it has been demonstrated that isoprene is crucial for maintaining electron transport rates under heat and drought stress episodes (Vanzo et al. 2015; Monson et al. 2020). Our data do not provide direct evidence for the functionality of isoprene in the mitigation of the effect of heat and drought stress on photosynthesis in date palm, which would require, e.g., a transgenic approach with non-isoprene emitting date palms. However, proteome analysis and correlation with isoprene emissions in this study suggest the interplay between the plastic and coordinated protein composition and biochemical processes involved in the protection and maintenance of central processes of primary plant metabolism.

### Identification and functional characterization of isoprene synthase in date palm

The trait to emit isoprene is characteristic to plant species with high growth rates and affinity for sunny environment (Harley et al. 1999; Loreto et al. 2015). To date, genes encoding isoprene synthase have been reported for a limited number of plant species (Sharkey et al. 2013). Although it has long been known that palm species emit isoprene (Benjamin et al. 1996), the gene responsible for this emission remained unknown. Here, we identified, reconstructed and experimentally validated a complete CDS sequence encoding a *IspS* in *P. dactylifera*, a monocotyledon species from the order Arecales. To this end, we performed a correlation analysis of annotated *P. dactylifera* peptide sequence abundances with the different treatment conditions, which allowed the identification of this enzyme from the potential terpene synthase (TPS)-like peptides. Our screening of peptide sequences with IspS homologous sequences from literature yielded three reasonable peptide candidates, but only one contained the first three residues of the diagnostic IspS tetrad F(V/S)F(N/S) (Sharkey et al. 2013; Li et al. 2017). Although mutagenesis experiments could prove the functional significance of all four residues of the diagnostic IspS tetrad (Li et al. 2017), the second and fourth residues in the date palm sequence show some variation among known IspS. Instead of asparagine (dicotyledonous IspS) or serine (monocotyledonous IspS), PdIspS contains threonine (T479) at the position of the fourth residue of the diagnostic tetrad. All three amino acids are polar and uncharged and can substitute each other in IspS. We focused our analysis on this unique protein, which showed a correlation with isoprene emission in summer climate, since we knew that the *IspS* promoter is activated by light (Cinege et al. 2009) and that the enzyme is upregulated under high temperature and seasonal conditions (Lehning et al. 1999; Mayrhofer et al. 2005). Among the functionally characterized plant IspS, PdIspS shares the highest amino acid sequence identity to the monocot *Arundo donax* AdIspS (52%; Li et al. 2017; but see Note added at the end of the paper). The metal-binding motifs DDXXD and DTE/NSE and RXR motif of the mature PdIspS clearly demonstrate that, like other IspS, PdIspS belongs to the class type I of the TPS family (Zhou and Pichersky 2020).

Finally, the functional analysis of the PdIspS protein expressed in *E. coli* proved that the gene XP_008779509.1 indeed encodes the IspS of date palm. Another typical feature of PdIspS is the very high temperature optimum of its catalytic activity (46.5 °C), which is characteristic for all IspS characterized so far (Silver and Fall 1991; Lehning et al. 1999; Schnitzler et al. 2005). This optimum also helps to explain the high emission rates of date palms under the climate conditions of the Arabian Peninsula. The present identification of *PdIspS* and the encoding enzyme will pave the way to study in more detail the isoprene functions in date palm under extreme environments.

### Heat shock protein and the antioxidant system responses to contrast ROS formation

Well-known mechanisms to cope with heat and drought stresses are the upregulation of Hsps and the enzymatic or non-enzymatic scavenging of ROS. Heat causes protein unfolding, and molecular chaperons are the first line of protection to detect misfolded proteins and prevent their aggregation in cells (Wang et al. 2004). In this respect, the 16 Hsps upregulated in date palm leaves acclimated to summer climate appear to play a crucial role in thermotolerance under heat by stabilizing membranes and protein motifs. The Hsps abundances increased further under water limitation, suggesting that the drought-induced stomatal closure exacerbated the temperature effects on leaves. As Hsps are normally induced upon pH shift or hypoxia (Al-Whaibi 2011; Ul Haq et al. 2019), the accumulation of molecular chaperons observed in this study confirmed that the leaves experienced stressful conditions. However, the proteasome, the primary proteolytic system involved in the removal of oxidatively damaged proteins, was neither upregulated in summer conditions compared to winter conditions nor under summer drought and compared to well-watered plants, suggesting that there was no need to employ the degradation machinery to remove denatured proteins. Interestingly, the abundance of proteins involved in proteolysis was lower in summer compared to winter climate, when isoprene emission and the proteins involved in abiotic stress response were high. Their abundances increased under soil water deprivation in summer when isoprene decreased, but remained unchanged under drought in winter concomitant to unchanged isoprene emissions. This observation points to a diverse mechanism to counteract drought stress under winter and summer climate, possibly involving isoprene or other molecules closely related to photosynthetic supply limitation. Taken together, the results suggest a multifaceted mechanism in summer and winter climate to contrast oxidative stress and efficiently avoid protein damage.

In general, heat and drought cause oxidative stress and produce ROS (Sharma et al. 2020). Increasing levels of ROS function as a signaling mechanism to activate a series of acclamatory and protective responses mainly via hydroxyl radicals. Excess of ROS is dangerous for the plant cells as ROS can oxidize a series of molecules (protein, lipids, DNA), leading to cellular dysfunction and eventually, cell death. Since we did not observe any phenotypic effects under summer climate or under drought, the ROS formation was counteracted by an adequate antioxidant response via non-enzymatic and enzymatic mechanisms. Particularly noteworthy was the upregulation of the plastid SOD and APX involved in the Foyer-Halliwell-Asada cycle. However, we also observed downregulation of the GR protein. The lower level of GR that catalyzes the reduction of glutathione disulfide to its sulfhydryl form of tripeptide glutathione may have been offset by increased enzyme activity, as shown by an in vitro assay from a previous date palm experiment (Arab et al. 2016). Interestingly, heat stress often leads to the inactivation of methionine sulfoxide by oxidation (Davies 2005). The date palm seemed to compensate for this by upregulating the expression of MSR, the reductase that restores protein activity by catalyzing the reduction of methionine sulfoxide to methionine.

It is worth noting that the increasing enzyme activities of the antioxidant system (Arab et al. 2016) coincide with the remarkable upregulation of the antioxidant and Hsps proteins. Their close connection agrees with a feedback loop regulation to maintain homeostasis, as ROS activate the expression of Hsps, and the formation of Hsps can enhance the enzyme activities of the antioxidant system, including POD, CAT and SOD, which in turn reduce ROS formation (Driedonks et al. 2015; Ul Haq et al. 2019). Also, non-enzymatic reactions occur in cells between ROS and phenolic compounds such as phenylpropanoids and flavonoids that may act as protective molecules against oxidative stress by scavenging radical formation and prevent lipid peroxidation (Agati and Tattini 2010; Mierziak et al. 2014). Our study shows a decreased expression of proteins involved in the biosynthesis of these secondary metabolites under higher temperature and drought conditions (except for isoprene). This confirms that the upregulation of the Hsps and the antioxidant system was efficient in counteracting ROS formation under drought and with the help of isoprene formation under higher temperatures. In adition, it suggests that investing in protein changes and isoprene biosynthesis instead of non-volatile secondary compounds is a successful strategy of date palm to cope with heat and drought.

Taking together, we conclude that date palm evolved a complex multi-mechanism based on increasing abundances of proteins involved in abiotic stress defense (Hsps) and redox homeostasis (Fig. 5) and isoprene production to counteract the stress mediated by summer temperature conditions and soil aridity of the Arabian Peninsula.

## Supplementary Information

Below is the link to the electronic supplementary material.

Supplementary file1 (PDF 965 kb)

Supplementary file2 (Table S2) (XLSX 195 kb)
