# Supplementary material for: Protein expression plasticity contributes to heat and drought tolerance of date palm
Source: Oecologia. 2021 Apr 21;197(4):903–19. doi: 10.1007/s00442-021-04907-w (PMC8591023; doi:10.1007/s00442-021-04907-w)
Supplement: Supplementary file 1 — Supplementary file1 (PDF 965 kb) [file 442_2021_4907_MOESM1_ESM.pdf]

Supplementary Information:

Article title: **Protein expression plasticity contributes to heat and drought tolerance of date palm**

Authors: Andrea Ghirardo, Tetyana Nosenko, Jürgen Kreuzwieser, J. Barbro Winkler, Jörg Kruse, Andreas Albert, Juliane Merl-Pham, Thomas Lux, Peter Ache, Ina Zimmer, Saleh Alfarraj, Klaus F. X. Mayer, Rainer Hedrich, Heinz Rennenberg, Jörg-Peter Schnitzler

The following Supplementary Information is available for this article:

Material and Methods MM1: Reconstruction of the *PdIspS* CDS sequence
Material and Methods MM2: *In vitro* enzymatic activity assay of *P. dactylifera IspS* following expression in *E. coli* and protein purification

Figure S1. Daily environmental conditions
Figure S2. Effects of climate and drought on fresh weight and dry to fresh weight ratio of date palm leaves
Figure S3. Comparison of consensus PdIspS protein sequence to functionally characterized plant isoprene synthases
Figure S4. Consensus *PdIspS* CDS
Figure S5. *PdIspS* protein sequence

Table S1. VOC emitted from date palm leaves and log2 of fold changes between treatments
Table S2. Proteomic data (*see separate excel file*)

Material and Methods MM1: Reconstruction of the *PdIspS* CDS sequence

To reconstruct the N- and C-termini of the partial *PdIspS* sequence XP_008779509.1, we identified close orthologs of this gene from other monocots from the NCBInr database (ASF20076.1 and XP_010923912.1 from *Arundo donax* and *Elaeis guineensis*, respectively) and searched genome sequence data for two *P. dactylifera* varieties (NCBI; Bioprojects PRJNA396270 and PRJNA322046) using terminal parts of these genes. Three contigs from the Khanizi cultivar (PEFZ01192174.1, PEFZ01199267.1, and PEFZ01103933.1) and two unplaced scaffolds from the Khalas cultivar (NW_008252718.1 and NW_008252718.1) resulting from this search were assembled in a single pseudo-scaffold. A complete CDS of the *P. dactylifera* putative *IspS* gene was reconstructed based on the RNA-seq reads aligned to this pseudoscaffold. Briefly, to identify samples in which XP_008779509.1 is expressed, RNA-seq data for the *P. dactilyfera* leaf tissue from the Bioprojects PRJEB18007 and PRJEB22923 were obtained from NCBI. All transcript models predicted for the Khalas genome assembly have been indexed and transcript abundance in the aforementioned RNA-seq libraries were quantified using Kallisto v0.44.0 (Bray et al. 2016) using default parameters. Libraries characterized by a high abundance of the XP_008779509.1 transcripts were selected for further analysis. RNA-seq reads were filtered, quality trimmed using Trimmomatic v.0.35 (Bolger et al. 2014), and mapped to the reference consisting of the *P. dactilyfera* genome assembly GCA_000413155.1 and the *IspS* pseudo-scaffold using STAR v2.5.2a (Dobin et al. 2013). The *IspS* pseudo-scaffold alignment region was extracted from the bam files using SAMtools (Li et al. 2009) and visualized and processed using Integrative Genomics Viewer v2.4.19 (Robinson et al. 2011). The *IspS* coding sequence (CDS) encoded by this pseudo-scaffold was reconstructed based on the consensus of RNA-seq sequences aligned to this region. The *IspS* intron-exon splice sites were defined manually based on the alignments. Nucleotide and amino acid CDS sequences of PdIspS are provided in the Supplementary Figs S3-5.

Material and Methods MM2: *In vitro* enzymatic activity assay of *P. dactylifera IspS* following expression in *E. coli* and protein purification

Date palm IspS activities were determined at 5mM of the substrate dimethylallyl diphosphate (DMADP) for isoprene biosynthesis or by 5 mM geranyl diphosphate (GDP) for monoterpenes. Each sample contained 50 µL protein extract, corresponding to 169 µg in the empty vector (EV) control and 174 µg in PdIspS samples, respectively, as determined by the Bradford assay. The incubation time was 30 min, and the standard incubation temperature was 40°C. For the temperature dependence analysis of PdIspS, samples were incubated between 20 and 60° with 5°C steps and the respective data collected from EV were used for background corrections. After incubation, the reaction was stopped by removal of the aqueous solution and rinsing the vial with 100 µL buffer solution. Finally, the headspace was injected by a self-made autosampler in the inlet of PTR-QMS using N_2 as carrier gas (170 ml min^{-1}) for VOC analysis. The PTR-QMS was operated at *E*/*N* = 132 Td, p-drift = 2.2 mbar, Tdrift = 60 °C, Udrift = 590V, O_2^+ and NO^+ < 2% of H_3O^+; $H_2OH_3O^+$ <8% of H_3O^+. Five replicates were analyzed for each sample. Quantitative analysis was achieved by the dilution curve of 11 VOC-mixture standards in N_2 (Apel-Riemer Environmental, Miami, FL, USA) containing isoprene and the monoterpene α-pinene, diluted between 0-640 ppbv (four-point calibration) by N_2 (5.0 purity) at 90% of rel. humidity and 24°C (r^2>0.98). For each concentration, standards were produced in triplicate. Calibration curves were performed before and after each analysis. No statistical difference was found between the pre and post-calibration procedures. Therefore, the mean of the instrumental sensitivities was used for final VOC quantification.

Supplementary Figures:

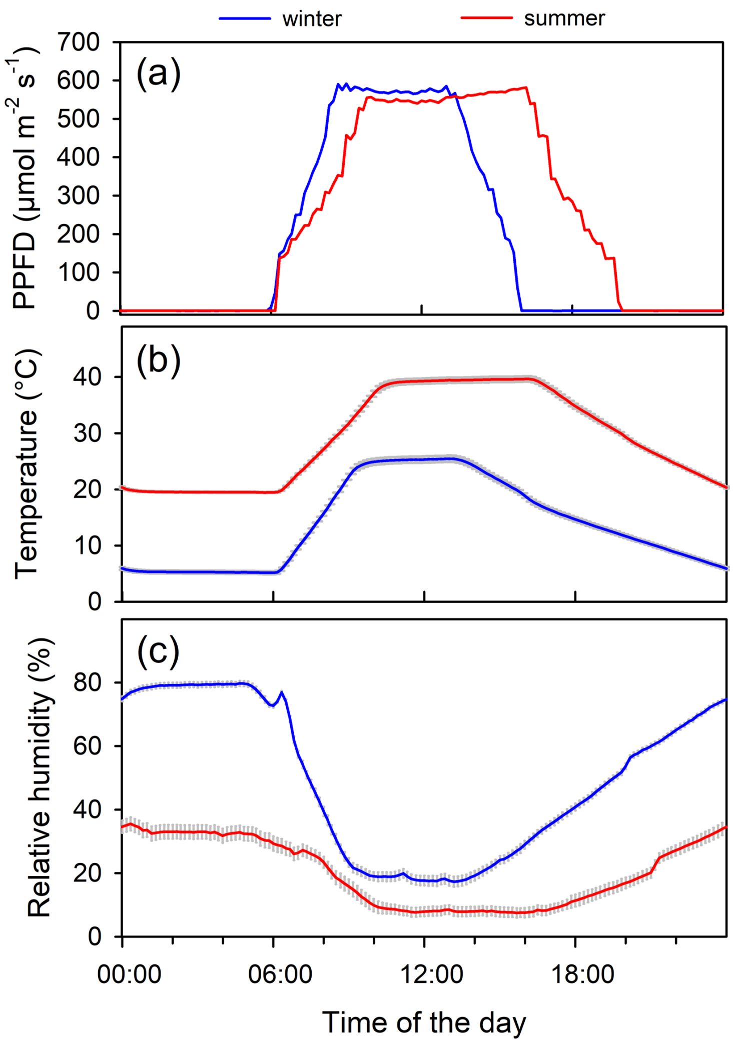

Figure S1. Daily environmental conditions

Daily environmental conditions measured during the simulation of winter (in blue) and summer average day (in red) in Saudi Arabia. (**a**) Diurnal variation of light (incident photosynthetically active quantum flux density, PPFD), (**b**) air temperature, and (**c**) relative humidity inside the climate chambers. The environmental conditions represent the mean values of the ten years climate (2003-2012) in Alahsa, Saudi-Arabia, in winter (21.12-21.03) or summer (21.06-21.09). These conditions were repeated throughout the entire experimental period. Data depict means of n = 4 sub-chambers, ± s.d.

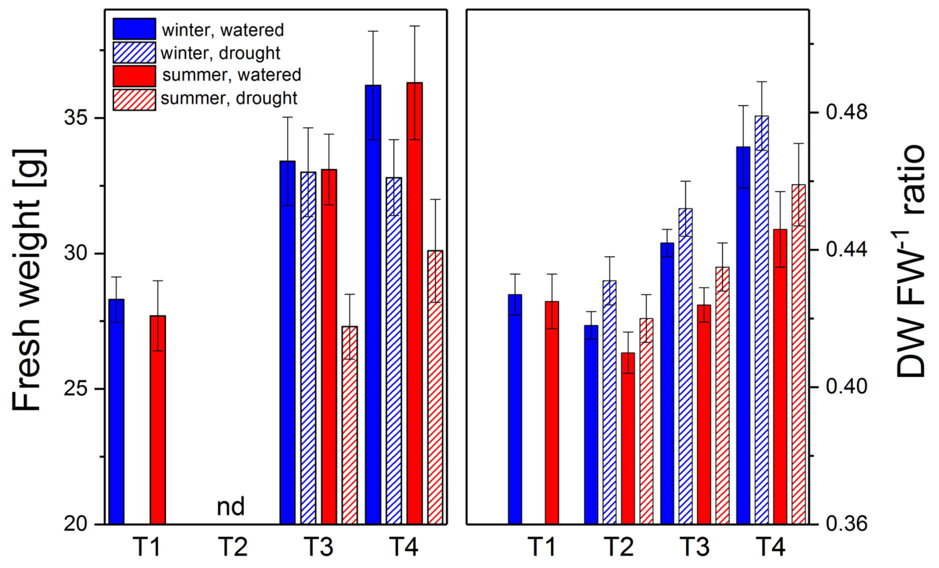

Figure S2. Effects of climate and drought on fresh weight and dry to fresh weight ratio (DW FW^{-1}) of date palm leaves.

Blue and red colors indicate data obtained from plants growing under winter and summer climate, respectively. Pattern bars refer to drought treatments. T1-T4 indicates sampling times under pre-stress (T1), mild (T2) and severe (T3) drought (T3), and post-drought stress recovery phase as a result of re-watering (T4). Data shown are means ± s.e. of 5 plants.

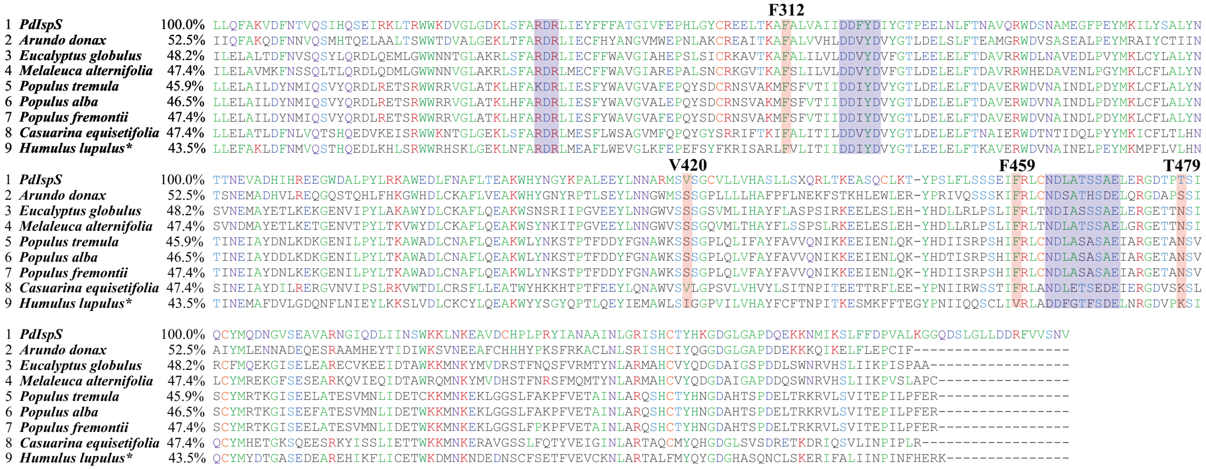

Figure S3. Comparison of consensus PdIspS protein sequence to functionally characterized plant isoprene synthases

Multiple sequence alignment of the C-terminal domain (Class I terpenoid synthase fold). Red shading indicates residues of the diagnostic tetrad. Numbers in bold refer to the positions in the amino acid sequence of the *PdIspS* protein precursor. The purple shading indicates conserved TPS motifs: RXR, DDXXD and DTE/NSE. Dots represent residues that are identical to those in *PdIspS.* A star indicates the *Humulus lupulus* myrcene synthase, which shows a minor *IspS* activity (Sharkey et al. 2013). NCBI accession numbers of genes included in the alignment are: *Arundo donax* ASF20076.1, *Eucalyptus globulus* BAF02831.1, *Melaleuca alternifolia* AAP40638.1, *Populus tremula* CAC35696, *P. alba* ADG96473.1, *P. fremontii* AEK70967.1, *Casuarina equisetifolia* BAS30549.1, and *Humulus lupulus* ACI32638.1.

<u>ATGGCACTCTCTACATGCTTCGCATCCACCGCATGTCATGCCGGGAGCAAAGGTT</u>
<u>GGGTGGTG</u>GCAAGTCAAGTCCGTTCCCAACCTCCGACGAGGCGATCGGCAAATTA
TCAGCCTAGCTCATGGGACTATGACTCCCTTCAGTCTTTGAGGGGTGGCGACTTG
AACGGACACACACGACACACTTGGAAAAGTTGAAGGAGAACACGAGGCACTTG
CTATCCAAGGAGGCTGAACCAGTGGCTCGACTTAAGCTGGTCGACGTCCTGCAGC
GTCTTGGTGTGGGTTATCACTTTGAGGAGGAGATCAAGGATGCCCTTGGCTCGAT
GCCCATTGAGAGGGCAAACACATTGTTCAAAGACGACATCCACTCCATGTCTTTG
CTATTTAGACTGCTTAGAGAGCACGGGTTTCCTGTTTCCCCAGACATCTTCAGTAG
CTTGAAAGAAGAGAACGGTAACTTCAAGGCAAGCCTTCTCAGGGACACTCAAGC
ATTATTATCCCTACATGAGGCTTCATATCTTGCTTTTGAAGGAGAAACACTGCTGG
ATGAAGCAAGAATTTTCACAACTAAATATCTCAGCGATCTCAAGCTACAGATGGA
TCCACACCTTAAGGGAAAAGTKTCTCTATCTTTGGATCTTCCTTTGCACTGGAGGA
CTCCAAGGTTGGAAGCAAGATGGCACATAGATCAGTATGAAAGAGATGGAAACA
TGGACCATAYGCTTCTTCAATTTGCTAAGGTCGATTTCAACACAGTGCAAAGCAT
ACACCAAAGTGAAATTAGAAAACTGACAAGGTGGTGGAAAGATGTAGGCCTTGG
GGATAAATTGAGCTTTGCTAGAGATCGATTGATAGAGTACTTCTTCTTTGCAACTG
GAATTGTATTCGAGCCGCATCTTGGATATTGTCGAGAGGAACTTACTAAAGCATT
TGCTTTGGTAGCAATTATAGATGATTTCTATGATATTTACGGAACACCGGAAGAG
CTTAACCTTTTCACCAAYGCCGTCCAGAGGTGGGACTCCAATGCAATGGAAGGCT
TTCCAGAGTAYATGAAAATCTTGTATTCAGCTTTGTACAACACAACYAATGAAGT
GGCRGATCACATACATAGAGAAGAGGGYTGGGATGCCCTTCCTTATCTCAGAAA
AGCGTGGGAGGACCTATTCAACGCCTTCCTAACGGAGGCAAAGTGGCATTACAA
CGGCTACAAACCAGCCCTCGAGGAGTATCTAAACAATGCYCGGATGTCAGTSTCA
GGCTGCGTCCTATTAGTCCATGCTAGCCTCTTATCAASGCAGAGACTAACYAAGG
AGGCATCGCAGTGCTTGAAGACCTATCCAAGCCTCTTCCTGTCCTCATCCGAGAT
CTTTCGTCTTTGCAATGATTTAGCCACTTCTTCGGCAGAGTTAGAGAGAGGTGATA
CTCCAACATCCATCCAATGCTACATGCAAGACAATGGTGTMTCCGAGGCAGTTGC
TCGCAATGGCATTCAAGATTTAATCATTAATTCATGGAAGAAGCTGAACAAAGAA
GCTGTTGATTGCCATCCACTCCCTCGATACATTGCTAATGCAGCCATAAATCTTGG
ACGGATATCGCATTGCACATACCATAAGGGAGATGGCTTGGGTGCTCCAGATCAA
GAGAAGAAGAATATGATCAAGTCCCTGTTTTTGATCCTGTAGCACTCAAAGGAG
GCCAGGAYTCTTTAGGGCTTCTTGATGATAGGTTTGTGGTCAGCAATGTGTAG

Figure S4. Consensus *PdIspS* CDS

The coding sequence of isoprene synthase in *Phoenix dactylifera* (*PdIspS*). The sequence encoding predicted plastid-targeted peptide is underlined.

<u>MALSTCFASTACHAGSKGWVV</u>ASQVRSQPPTRRSANYQPSSWDYDSLQSLRGGDLN
GTHTTHLEKLKENTRHLLSKEAEPVARLKLVDVLQRLGVGYHFEEEIKDALGSMPIE
RANTLFKDDIHSMSLLFRLLREHGFPVSPDIFSSLKEENGNFKASLLRDTQALLSLHEA
SYLAFEGETLLDEARIFTTKYLSDLKLQMDPHLKGKVSLSLDLPLHWRTPRLEARWHI
DQYERDGNMDHXLLQFAKVDFNTVQSIHQSEIRKLTRWWKDVGLGDKLSFARDRLI
EYFFFATGIVFEPHLGYCREELTKAFALVAIIDDFYDIYGTPEELNLFTNAVQRWDSNA
MEGFPEYMKILYSALYNTTNEVADHIHREEGWDALPYLRKAWEDLFNAFLTEAKWH
YNGYKPALEEYLNNARMSVSGCVLLVHASLLSXQRLTKEASQCLKTYPSLFLSSSEIF
RLCNDLATSSAELERGDTPTSIQCYMQDNGVSEAVARNGIQDLIINSWKKLNKEAVD
CHPLPRYIANAAINLGRISHCTYHKGDGLGAPDQEKKNMIKSLFFDPVALKGGQDSL
GLLDDRFVVSNV*

Figure S5. *PdIspS* protein sequence

Amino acid sequence of the isoprene synthase in *Phoenix dactylifera* (*PdIspS*). The predicted plastid-targeted peptide is underlined. The TargetP cleavage site probability was 0.722.

Supplementary Table:

Table S1. VOC emitted from date palm leaves and log2 of fold changes between treatments.

Volatiles were collected on VOC adsorbents from enclosed palm leaves under standard conditions (T= 30 °C; 400 ppm; 1,000 µmol m^{-2} s^{-1} PPFD) and analyzed by GC-MS analysis. No indicate compounds shown in Figure 2 (biplot of PCA). Increased and decreased emissions due to treatments are indicated by the intensity of red and blue colors, respectively. Significant differences between treatments at *p* <0.05 as calculated by non-parametric *t*-test are indicated in bold.

No	Compound	Summer/ Winter Control	Summer drought/ Control	Winter drought/ Control
1	Acetaldehyde	-0.47	-1.00	-0.59
2	Ethanol	0.21	-1.64	-3.76
3	Isoprene	0.54	-0.44	-0.08
4	1-Butene, 2-methyl-	-0.10	-2.21	**-3.30**
5	2-Propanone	0.33	-1.28	-1.88
6	3-Hexene, Z-	0.07	-2.27	**-2.57**
7	2-Hexene	0.08	-2.34	**-2.64**
8	2-Pentene, 3-methyl-, E-	0.06	-2.40	**-2.64**
9	Butane, 2,2,3-trimethyl-	0.28	-2.29	**-2.62**
10	Cyclopentane, methyl-	-0.32	0.18	0.64
11	Cyclohexane	-0.15	0.49	0.67
12	1-Pentene, 2,4,4-trimethyl-	-0.02	-0.28	-0.39
13	2-Pentene, 2,3,4-trimethyl-	0.45	-1.43	**-2.34**
14	1,2,4-Trimethyl-cyclopentane	-1.52	-0.94	0.34
15	trans-4,4-Dimethyl-2-hexene	0.07	-1.96	**-2.37**
16	Heptane, 2,4-dimethyl-	-0.41	-0.27	-0.08
17	1-Hexene, 4,5-dimethyl-	0.31	-0.85	-1.43
18	1-Hexene, 3,4-dimethyl-	0.33	-1.07	-1.82
19	Cyclohexane, 1-methyl-3-pentyl-	0.44	-0.25	-1.19
20	4-Undecanol, 7-ethyl-2-methyl-	-0.01	-0.84	-1.24
21	Benzene, 1-methyl-4-1-methylethyl-	0.08	-0.72	-1.34

Table S2. Proteomic data.
See separate excel file

References:

Bolger AM, Lohse M, Usadel B (2014) Trimmomatic: A flexible trimmer for Illumina sequence data. Bioinformatics 30:2114–2120. https://doi.org/10.1093/bioinformatics/btu170

Bray NL, Pimentel H, Melsted P, Pachter L (2016) Near-optimal probabilistic RNA-seq quantification. Nat Biotechnol 34:525–527. https://doi.org/10.1038/nbt.3519

Dobin A, Davis CA, Schlesinger F, et al (2013) STAR: Ultrafast universal RNA-seq aligner. Bioinformatics 29:15–21. https://doi.org/10.1093/bioinformatics/bts635

Ghirardo A, Koch K, Taipale R, et al (2010) Determination of de novo and pool emissions of terpenes from four common boreal/alpine trees by $^{13}CO_2$ labelling and PTR-MS analysis. Plant cell Environ 33:781–792. https://doi.org/10.1111/j.1365-3040.2009.02104.x

Ghirardo A, Wright LP, Bi Z, et al (2014) Metabolic flux analysis of plastidic isoprenoid biosynthesis in poplar leaves emitting and nonemitting isoprene. Plant Physiol 165:37–51. https://doi.org/10.1104/pp.114.236018

Li H, Handsaker B, Wysoker A, et al (2009) The Sequence Alignment/Map format and SAMtools. Bioinformatics 25:2078–2079. https://doi.org/10.1093/bioinformatics/btp352

Mayrhofer S, Teuber M, Zimmer I, et al (2005) Diurnal and seasonal variation of isoprene biosynthesis-related genes in grey poplar leaves. Plant Physiol 139:474–484. https://doi.org/10.1104/pp.105.066373.474

Robinson JT, Thorvaldsdóttir H, Winckler W, et al (2011) Integrative genomics viewer. Nat Biotechnol 29:24–26. https://doi.org/10.1038/nbt.1754

Schnitzler J-P, Zimmer I, Bachl A, et al (2005) Biochemical properties of isoprene synthase in poplar (*Populus* x *canescens*). Planta 222:777–786. https://doi.org/10.1007/s00425-005-0022-1
